# 6-Chloro-4-oxo-4*H*-chromene-3-carb­aldehyde

**DOI:** 10.1107/S1600536814007119

**Published:** 2014-04-05

**Authors:** Yoshinobu Ishikawa

**Affiliations:** a School of Pharmaceutical Sciences, University of Shizuoka, 52-1 Yada, Suruga-ku, Shizuoka 422-8526, Japan

## Abstract

In the title compound, C_10_H_5_ClO_3_, a chlorinated 3-formyl­chromone derivative, the non-H atoms are essentially coplanar (r.m.s. deviation = 0.0456 Å) with the largest deviation from the least-squares plane [0.1136 (16) Å] being found for the ring-bound carbonyl O atom. In the crystal, mol­ecules are linked through stacking inter­actions along the *b* axis [shortest centroid–centroid distance between the pyran and benzene rings = 3.4959 (15) Å].

## Related literature   

For related structures, see: Ishikawa & Motohashi (2013 ▶); Ishikawa (2014 ▶). For van der Waals radii; see: Bondi (1964 ▶). For halogen bonding, see: Auffinger *et al.* (2004 ▶); Metrangolo *et al.* (2005 ▶); Sirimulla *et al.* (2013 ▶).
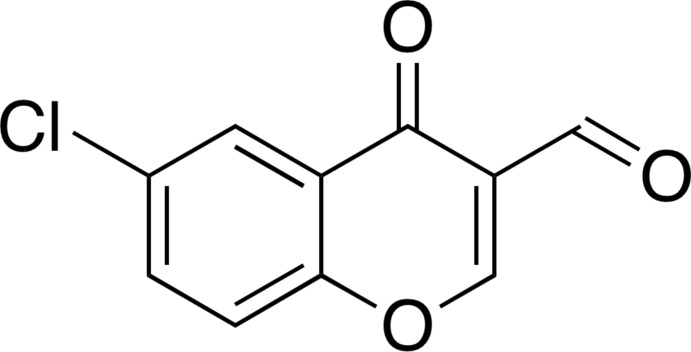


## Experimental   

### 

#### Crystal data  

C_10_H_5_ClO_3_
*M* *_r_* = 208.60
Triclinic, 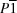

*a* = 6.5838 (16) Å
*b* = 6.9579 (17) Å
*c* = 10.265 (3) Å
α = 71.22 (3)°
β = 85.64 (2)°
γ = 69.29 (3)°
*V* = 416.0 (2) Å^3^
*Z* = 2
Mo *K*α radiation
μ = 0.43 mm^−1^
*T* = 100 K
0.36 × 0.25 × 0.12 mm

#### Data collection  

Rigaku AFC-7R diffractometer
Absorption correction: ψ scan (North *et al.*, 1968 ▶) *T*_min_ = 0.891, *T*_max_ = 0.950
2356 measured reflections
1906 independent reflections
1741 reflections with *F* ^2^ > 2σ(*F* ^2^)
*R*_int_ = 0.058
3 standard reflections every 150 reflections intensity decay: −0.9%

#### Refinement  

*R*[*F* ^2^ > 2σ(*F* ^2^)] = 0.040
*wR*(*F* ^2^) = 0.109
*S* = 1.10
1906 reflections
127 parameters
H-atom parameters constrained
Δρ_max_ = 0.29 e Å^−3^
Δρ_min_ = −0.64 e Å^−3^

### 

Data collection: *WinAFC Diffractometer Control Software* (Rigaku, 1999 ▶); cell refinement: *WinAFC Diffractometer Control Software*; data reduction: *WinAFC Diffractometer Control Software*; program(s) used to solve structure: *SIR88* (Burla *et al.*, 1989 ▶); program(s) used to refine structure: *SHELXL97* (Sheldrick, 2008 ▶); molecular graphics: *CrystalStructure* (Rigaku, 2010 ▶); software used to prepare material for publication: *CrystalStructure*.

## Supplementary Material

Crystal structure: contains datablock(s) General, I. DOI: 10.1107/S1600536814007119/tk5303sup1.cif

Structure factors: contains datablock(s) I. DOI: 10.1107/S1600536814007119/tk5303Isup2.hkl

Supporting information file. DOI: 10.1107/S1600536814007119/tk5303Isup3.cml

CCDC reference: 994454

Additional supporting information:  crystallographic information; 3D view; checkCIF report

## Figures and Tables

**Fig. 1.**
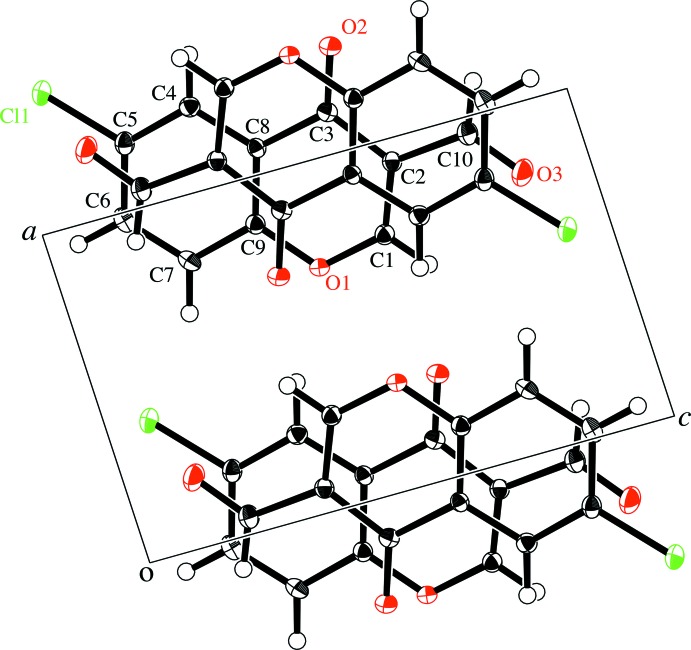
A packing view of the title compound, with displacement ellipsoids drawn at the 50% probability level. Hydrogen atoms are shown as small spheres of arbitrary radius.

**Fig. 2.**
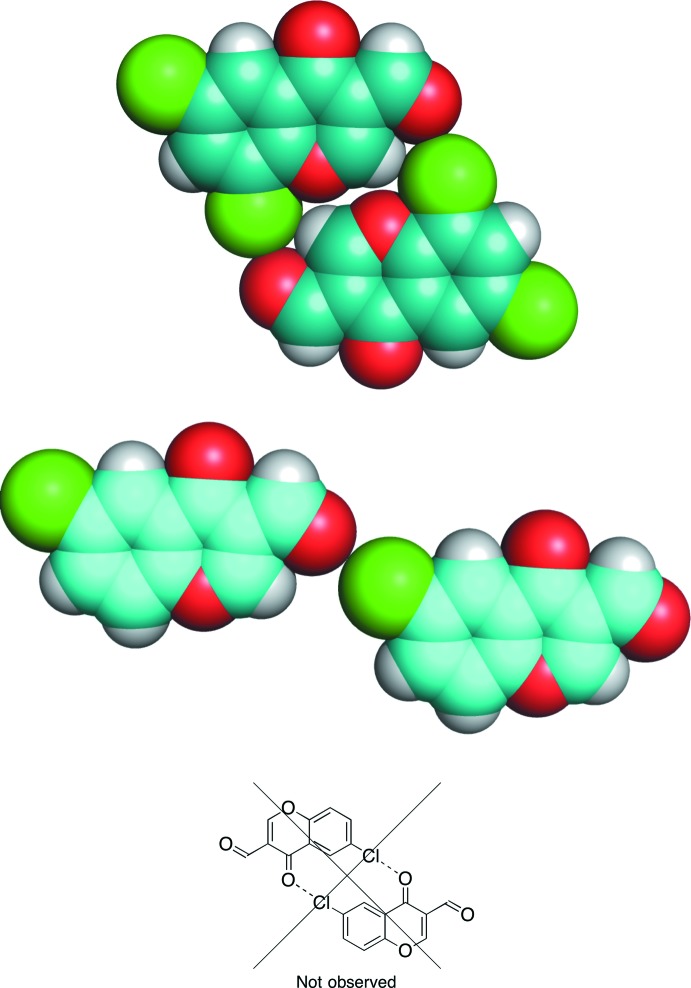
Sphere models of the crystal structures of 6,8-dichloro-4-oxochromene-3-carbaldehyde (top) and the title compound (middle), and an illustration of a hypothetical model of the title compound with halogen bonds (bottom).

## supplementary crystallographic information

### 1. Structural commentary

Halogen bonds have been found to occur in organic, inorganic and biological systems, and have recently attracted much attention in medicinal chemistry, chemical biology and supra­molecular chemistry (Auffinger *et al.*, 2004; Metrangolo *et al.*, 2005; Sirimulla *et al.*, 2013). We have recently reported the crystal structures of dihalogenated 3-formyl­chromone derivatives 6,8-di­chloro-4-oxochromene-3-carbaldehyde (Ishikawa & Motohashi, 2013; Fig. 2 (top)) and 6,8-di­bromo-4-oxochromene-3-carbaldehyde (Ishikawa, 2014). It was found that similar halogen bonds between the formyl oxygen atom and the halogen atoms at the 8-position are formed in those crystal structures. As part of our inter­est in this type of chemical bonding, we herein report the crystal structure of a monochlorinated 3-formyl­chromone derivative, 6-chloro-4-oxo-4*H*-chromene-3-carbaldehyde. The objective of this study is to reveal whether halogen bond(s) can be formed in the crystal structure of this compound without halogen atom at 8-position.

The mean deviation of the least-square planes for the non-hydrogen atoms is 0.0456 Å, and the largest deviations is 0.1136 (16) Å for O2. These mean that these atoms are essentially coplanar.

In the crystal, the molecules are stacked with the inversion-symmetry equivalents along the *b*-axis direction [centroid–centroid distance between the pyran rings of the 4*H*-chromene units = 3.926 (2) Å, *i*: -*x* + 2, -*y* + 1, -*z* + 1], as shown in Fig. 1. The distance between the chloride atom and the formyl oxygen atom of the translation-symmetry equivalent [Cl1···O3^ii^ = 3.284 (2) Å, *ii*: *x* + 1, *y*, *z* - 1] is approximately equal to the sum of their van der Waals radii [3.27 Å] (Bondi, 1964), as shown in the middle of Fig. 2. Thus, it is concluded that there is no halogen bond in the title compound. The C–Cl···O and Cl···O=C angles are 166.30 (8) and 166.69 (14)°, respectively. The latter angle is greater than that of 6,8-di­chloro-4-oxochromene-3-carbaldehyde (Ishikawa & Motohashi, 2013). A structure with halogen bonds can be modeled for the title compound (Fig.2, bottom), but it is not observed in the crystal. These results might be invaluable for the development of state-of-the-art force fields.

### 2. Synthesis and crystallization

Single crystals suitable for X-ray diffraction were obtained by slow evaporation of an ethyl acetate solution of the commercially available title compound at room temperature.

### 3. Refinement

The C(sp^2^)-bound hydrogen atoms were placed in geometrical positions [C–H 0.95 Å, *U*_iso_(H) = 1.2*U*_eq_(C)], and refined using a riding model. One reflection (1 8 2) was omitted because of systematic error.

### Crystal data

**Table:**

C_10_H_5_ClO_3_	*Z* = 2
*M**_r_* = 208.60	*F*(000) = 212.00
Triclinic, *P*1	*D*_x_ = 1.665 Mg m^−^^3^
Hall symbol: -P 1	Mo *K*α radiation, λ = 0.71069 Å
*a* = 6.5838 (16) Å	Cell parameters from 25 reflections
*b* = 6.9579 (17) Å	θ = 15.3–17.2°
*c* = 10.265 (3) Å	µ = 0.43 mm^−^^1^
α = 71.22 (3)°	*T* = 100 K
β = 85.64 (2)°	Plate, colourless
γ = 69.29 (3)°	0.36 × 0.25 × 0.12 mm
*V* = 416.0 (2) Å^3^	

### Data collection

**Table:**

Rigaku AFC-7R diffractometer	*R*_int_ = 0.058
ω–2θ scans	θ_max_ = 27.5°
Absorption correction: ψ scan (North *et al.*, 1968)	*h* = −4→8
*T*_min_ = 0.891, *T*_max_ = 0.950	*k* = −8→9
2356 measured reflections	*l* = −13→13
1906 independent reflections	3 standard reflections every 150 reflections
1741 reflections with *F*^2^ > 2σ(*F*^2^)	intensity decay: −0.9%

### Refinement

**Table:**

Refinement on *F*^2^	Secondary atom site location: difference Fourier map
*R*[*F*^2^ > 2σ(*F*^2^)] = 0.040	Hydrogen site location: inferred from neighbouring sites
*wR*(*F*^2^) = 0.109	H-atom parameters constrained
*S* = 1.10	*w* = 1/[σ^2^(*F*_o_^2^) + (0.0651*P*)^2^ + 0.1805*P*] where *P* = (*F*_o_^2^ + 2*F*_c_^2^)/3
1906 reflections	(Δ/σ)_max_ < 0.001
127 parameters	Δρ_max_ = 0.29 e Å^−^^3^
0 restraints	Δρ_min_ = −0.64 e Å^−^^3^
Primary atom site location: structure-invariant direct methods	

### Special details

**Table:**

Refinement. Refinement was performed using all reflections. The weighted *R*-factor (*wR*) and goodness of fit (*S*) are based on *F*^2^. *R*-factor (gt) are based on *F*. The threshold expression of *F*^2^ > 2.0 σ(*F*^2^) is used only for calculating *R*-factor (gt).

### Fractional atomic coordinates and isotropic or equivalent isotropic displacement parameters (Å^2^)

**Table:**

	*x*	*y*	*z*	*U*_iso_*/*U*_eq_	
Cl1	1.39536 (6)	0.20927 (6)	0.08023 (4)	0.01987 (16)	
O1	0.69076 (17)	0.31098 (18)	0.46559 (11)	0.0144 (3)	
O2	1.29992 (18)	0.1926 (2)	0.61081 (12)	0.0182 (3)	
O3	0.7968 (2)	0.2432 (3)	0.87417 (12)	0.0239 (3)	
C1	0.7314 (3)	0.2807 (3)	0.59818 (15)	0.0136 (3)	
C2	0.9290 (3)	0.2401 (3)	0.65261 (15)	0.0131 (3)	
C3	1.1186 (3)	0.2214 (3)	0.56792 (15)	0.0126 (3)	
C4	1.2409 (3)	0.2179 (3)	0.33066 (15)	0.0139 (3)	
C5	1.1898 (3)	0.2441 (3)	0.19633 (15)	0.0148 (3)	
C6	0.9759 (3)	0.2980 (3)	0.15125 (16)	0.0166 (4)	
C7	0.8106 (3)	0.3239 (3)	0.24213 (16)	0.0159 (4)	
C8	1.0736 (3)	0.2431 (3)	0.42402 (15)	0.0120 (3)	
C9	0.8618 (3)	0.2926 (3)	0.37834 (15)	0.0129 (3)	
C10	0.9496 (3)	0.2130 (3)	0.80066 (16)	0.0169 (4)	
H1	0.6135	0.2881	0.6580	0.0163*	
H2	1.3869	0.1834	0.3594	0.0167*	
H3	0.9443	0.3168	0.0582	0.0200*	
H4	0.6643	0.3625	0.2123	0.0191*	
H5	1.0911	0.1694	0.8402	0.0202*	

### Atomic displacement parameters (Å^2^)

**Table:**

	*U*^11^	*U*^22^	*U*^33^	*U*^12^	*U*^13^	*U*^23^
Cl1	0.0190 (3)	0.0257 (3)	0.0136 (3)	−0.00517 (16)	0.00438 (14)	−0.00820 (15)
O1	0.0101 (5)	0.0183 (6)	0.0134 (5)	−0.0037 (4)	−0.0001 (4)	−0.0044 (4)
O2	0.0125 (5)	0.0268 (6)	0.0158 (6)	−0.0066 (5)	−0.0007 (4)	−0.0074 (5)
O3	0.0222 (6)	0.0358 (8)	0.0180 (6)	−0.0114 (6)	0.0065 (5)	−0.0141 (6)
C1	0.0137 (7)	0.0133 (7)	0.0130 (7)	−0.0036 (6)	0.0010 (5)	−0.0043 (6)
C2	0.0133 (7)	0.0141 (7)	0.0124 (7)	−0.0041 (6)	0.0004 (6)	−0.0056 (6)
C3	0.0127 (7)	0.0115 (7)	0.0136 (7)	−0.0034 (6)	−0.0002 (6)	−0.0047 (6)
C4	0.0137 (7)	0.0133 (7)	0.0142 (7)	−0.0039 (6)	0.0003 (6)	−0.0044 (6)
C5	0.0176 (8)	0.0133 (7)	0.0132 (7)	−0.0047 (6)	0.0022 (6)	−0.0049 (6)
C6	0.0220 (8)	0.0154 (7)	0.0118 (7)	−0.0064 (6)	−0.0017 (6)	−0.0031 (6)
C7	0.0160 (7)	0.0164 (7)	0.0142 (7)	−0.0050 (6)	−0.0042 (6)	−0.0029 (6)
C8	0.0132 (7)	0.0110 (7)	0.0111 (7)	−0.0037 (6)	−0.0001 (6)	−0.0031 (5)
C9	0.0136 (7)	0.0119 (7)	0.0124 (7)	−0.0038 (6)	0.0005 (6)	−0.0033 (6)
C10	0.0189 (8)	0.0189 (8)	0.0140 (7)	−0.0066 (6)	0.0010 (6)	−0.0068 (6)

### Geometric parameters (Å, º)

**Table:**

Cl1—C5	1.7377 (17)	C4—C8	1.405 (3)
O1—C1	1.341 (2)	C5—C6	1.397 (3)
O1—C9	1.3802 (19)	C6—C7	1.379 (3)
O2—C3	1.230 (3)	C7—C9	1.393 (3)
O3—C10	1.210 (2)	C8—C9	1.392 (3)
C1—C2	1.354 (3)	C1—H1	0.950
C2—C3	1.457 (3)	C4—H2	0.950
C2—C10	1.481 (3)	C6—H3	0.950
C3—C8	1.478 (3)	C7—H4	0.950
C4—C5	1.383 (3)	C10—H5	0.950
			
O1···C3	2.866 (3)	C4···H3	3.2782
O2···C1	3.578 (3)	C5···H4	3.2649
O2···C4	2.872 (3)	C6···H2	3.2829
O2···C10	2.898 (3)	C8···H4	3.2890
O3···C1	2.812 (3)	C9···H1	3.1907
C1···C7	3.582 (3)	C9···H2	3.2715
C1···C8	2.759 (3)	C9···H3	3.2493
C2···C9	2.769 (3)	C10···H1	2.5487
C4···C7	2.807 (3)	H1···H5	3.4825
C5···C9	2.747 (3)	H3···H4	2.3384
C6···C8	2.795 (3)	Cl1···H3^xi^	3.1849
Cl1···O3^i^	3.2840 (16)	Cl1···H4^vi^	2.9565
O1···O1^ii^	3.1591 (18)	Cl1···H4^xi^	3.4118
O1···O2^iii^	3.1063 (19)	Cl1···H5^x^	3.4268
O1···O2^iv^	3.309 (3)	Cl1···H5^vii^	3.4313
O1···C1^ii^	3.118 (2)	O1···H1^ii^	2.7492
O1···C3^v^	3.589 (3)	O1···H2^iii^	2.8618
O1···C4^v^	3.484 (3)	O1···H2^v^	3.5281
O1···C8^v^	3.432 (2)	O2···H1^vi^	2.5039
O2···O1^vi^	3.1063 (19)	O2···H2^vii^	2.6366
O2···O1^iv^	3.309 (3)	O3···H3^ix^	2.4552
O2···C1^vi^	3.096 (3)	O3···H3^v^	3.5089
O2···C1^iv^	3.543 (3)	O3···H4^ii^	3.2371
O2···C4^vii^	3.274 (2)	O3···H5^xii^	3.2852
O2···C9^iv^	3.394 (3)	C1···H1^ii^	3.4760
O3···Cl1^viii^	3.2840 (16)	C1···H2^v^	3.4704
O3···C4^iv^	3.590 (3)	C3···H2^vii^	3.4084
O3···C5^iv^	3.436 (3)	C4···H1^v^	3.2703
O3···C6^ix^	3.339 (3)	C4···H4^vi^	3.3076
C1···O1^ii^	3.118 (2)	C5···H1^v^	3.3113
C1···O2^iii^	3.096 (3)	C5···H3^xi^	3.2003
C1···O2^iv^	3.543 (3)	C5···H4^vi^	3.5285
C1···C4^v^	3.247 (3)	C6···H3^xi^	3.0323
C1···C5^v^	3.452 (3)	C6···H5^x^	3.5441
C1···C8^v^	3.487 (3)	C6···H5^v^	3.4065
C2···C4^iv^	3.592 (3)	C7···H1^ii^	3.4852
C2···C5^v^	3.562 (3)	C7···H2^iii^	3.2936
C2···C6^v^	3.485 (3)	C9···H1^ii^	3.3852
C2···C7^v^	3.507 (3)	C9···H2^iii^	3.5013
C2···C8^iv^	3.418 (3)	C10···H3^ix^	2.9512
C2···C9^v^	3.580 (3)	C10···H3^v^	3.3274
C3···O1^v^	3.589 (3)	H1···O1^ii^	2.7492
C3···C3^iv^	3.461 (3)	H1···O2^iii^	2.5039
C3···C7^v^	3.541 (3)	H1···C1^ii^	3.4760
C3···C8^iv^	3.512 (3)	H1···C4^v^	3.2703
C3···C9^v^	3.395 (3)	H1···C5^v^	3.3113
C4···O1^v^	3.484 (3)	H1···C7^ii^	3.4852
C4···O2^vii^	3.274 (2)	H1···C9^ii^	3.3852
C4···O3^iv^	3.590 (3)	H1···H1^ii^	3.5933
C4···C1^v^	3.247 (3)	H1···H2^v^	3.3433
C4···C2^iv^	3.592 (3)	H1···H4^ii^	3.0973
C4···C10^iv^	3.518 (3)	H2···O1^vi^	2.8618
C5···O3^iv^	3.436 (3)	H2···O1^v^	3.5281
C5···C1^v^	3.452 (3)	H2···O2^vii^	2.6366
C5···C2^v^	3.562 (3)	H2···C1^v^	3.4704
C5···C10^v^	3.600 (3)	H2···C3^vii^	3.4084
C5···C10^iv^	3.568 (3)	H2···C7^vi^	3.2936
C6···O3^x^	3.339 (3)	H2···C9^vi^	3.5013
C6···C2^v^	3.485 (3)	H2···H1^v^	3.3433
C6···C6^xi^	3.536 (3)	H2···H2^vii^	3.2073
C6···C10^v^	3.287 (3)	H2···H4^vi^	2.6733
C7···C2^v^	3.507 (3)	H3···Cl1^xi^	3.1849
C7···C3^v^	3.541 (3)	H3···O3^x^	2.4552
C8···O1^v^	3.432 (2)	H3···O3^v^	3.5089
C8···C1^v^	3.487 (3)	H3···C5^xi^	3.2003
C8···C2^iv^	3.418 (3)	H3···C6^xi^	3.0323
C8···C3^iv^	3.512 (3)	H3···C10^x^	2.9512
C8···C9^v^	3.512 (3)	H3···C10^v^	3.3274
C9···O2^iv^	3.394 (3)	H3···H3^xi^	2.7733
C9···C2^v^	3.580 (3)	H3···H5^x^	2.7277
C9···C3^v^	3.395 (3)	H3···H5^v^	3.2917
C9···C8^v^	3.512 (3)	H4···Cl1^iii^	2.9565
C10···C4^iv^	3.518 (3)	H4···Cl1^xi^	3.4118
C10···C5^v^	3.600 (3)	H4···O3^ii^	3.2371
C10···C5^iv^	3.568 (3)	H4···C4^iii^	3.3076
C10···C6^v^	3.287 (3)	H4···C5^iii^	3.5285
Cl1···H2	2.8098	H4···H1^ii^	3.0973
Cl1···H3	2.8018	H4···H2^iii^	2.6733
O1···H4	2.5183	H5···Cl1^ix^	3.4268
O2···H2	2.6171	H5···Cl1^vii^	3.4313
O2···H5	2.6235	H5···O3^xii^	3.2852
O3···H1	2.4841	H5···C6^ix^	3.5441
C1···H5	3.2768	H5···C6^v^	3.4065
C3···H1	3.2936	H5···H3^ix^	2.7277
C3···H2	2.6894	H5···H3^v^	3.2917
C3···H5	2.7009		
			
C1—O1—C9	118.51 (13)	C4—C8—C9	118.86 (15)
O1—C1—C2	124.50 (14)	O1—C9—C7	116.07 (15)
C1—C2—C3	120.89 (15)	O1—C9—C8	122.03 (15)
C1—C2—C10	118.68 (14)	C7—C9—C8	121.89 (15)
C3—C2—C10	120.43 (15)	O3—C10—C2	123.81 (16)
O2—C3—C2	123.55 (15)	O1—C1—H1	117.750
O2—C3—C8	122.50 (14)	C2—C1—H1	117.752
C2—C3—C8	113.94 (14)	C5—C4—H2	120.520
C5—C4—C8	118.97 (15)	C8—C4—H2	120.513
Cl1—C5—C4	119.56 (13)	C5—C6—H3	120.086
Cl1—C5—C6	118.90 (13)	C7—C6—H3	120.081
C4—C5—C6	121.54 (15)	C6—C7—H4	120.563
C5—C6—C7	119.83 (16)	C9—C7—H4	120.557
C6—C7—C9	118.88 (16)	O3—C10—H5	118.090
C3—C8—C4	121.17 (15)	C2—C10—H5	118.097
C3—C8—C9	119.97 (14)		
			
C1—O1—C9—C7	179.49 (13)	C8—C4—C5—Cl1	−178.97 (13)
C1—O1—C9—C8	0.2 (2)	C8—C4—C5—C6	1.0 (3)
C9—O1—C1—C2	2.4 (3)	H2—C4—C5—Cl1	1.0
C9—O1—C1—H1	−177.6	H2—C4—C5—C6	−179.0
O1—C1—C2—C3	−1.4 (3)	H2—C4—C8—C3	1.3
O1—C1—C2—C10	179.14 (13)	H2—C4—C8—C9	−179.7
H1—C1—C2—C3	178.6	Cl1—C5—C6—C7	179.23 (11)
H1—C1—C2—C10	−0.9	Cl1—C5—C6—H3	−0.8
C1—C2—C3—O2	177.29 (15)	C4—C5—C6—C7	−0.7 (3)
C1—C2—C3—C8	−2.0 (2)	C4—C5—C6—H3	179.3
C1—C2—C10—O3	−6.6 (3)	C5—C6—C7—C9	−0.8 (3)
C1—C2—C10—H5	173.4	C5—C6—C7—H4	179.1
C3—C2—C10—O3	173.91 (15)	H3—C6—C7—C9	179.1
C3—C2—C10—H5	−6.1	H3—C6—C7—H4	−0.9
C10—C2—C3—O2	−3.3 (3)	C6—C7—C9—O1	−177.08 (14)
C10—C2—C3—C8	177.49 (13)	C6—C7—C9—C8	2.2 (3)
O2—C3—C8—C4	4.1 (3)	H4—C7—C9—O1	2.9
O2—C3—C8—C9	−174.93 (14)	H4—C7—C9—C8	−177.8
C2—C3—C8—C4	−176.66 (13)	C3—C8—C9—O1	−3.7 (3)
C2—C3—C8—C9	4.3 (2)	C3—C8—C9—C7	177.13 (13)
C5—C4—C8—C3	−178.70 (13)	C4—C8—C9—O1	177.30 (14)
C5—C4—C8—C9	0.3 (3)	C4—C8—C9—C7	−1.9 (3)

Symmetry codes: (i) *x*+1, *y*, *z*−1; (ii) −*x*+1, −*y*+1, −*z*+1; (iii) *x*−1, *y*, *z*; (iv) −*x*+2, −*y*+1, −*z*+1; (v) −*x*+2, −*y*, −*z*+1; (vi) *x*+1, *y*, *z*; (vii) −*x*+3, −*y*, −*z*+1; (viii) *x*−1, *y*, *z*+1; (ix) *x*, *y*, *z*+1; (x) *x*, *y*, *z*−1; (xi) −*x*+2, −*y*+1, −*z*; (xii) −*x*+2, −*y*, −*z*+2.
